# Multi-objective inventory optimization using reinforcement learning: a comparative study on profitability and carbon emissions

**DOI:** 10.1038/s41598-026-44293-y

**Published:** 2026-04-28

**Authors:** Abdulrahman Sorour, Yomna Sadek, Mohamed Elshalakani

**Affiliations:** 1 Design and Production Engineering Department, Faculty of Engineering, Ain Shams University, 1 El Sarayat St., Abbasseya, El Weili, 11535 Cairo Governorate Egypt; 2 Mechatronics Engineering Department, Faculty of Engineering, Ain Shams University, 1 El Sarayat St., Abbasseya, El Weili, 11535 Cairo Governorate Egypt

**Keywords:** Inventory management, Reinforcement learning, Multi-objective optimization, Sustainability, Markov decision process (MDP), Engineering, Environmental sciences, Environmental social sciences, Mathematics and computing

## Abstract

Inventory management is a core part of supply chains, and over the years it has been increasingly challenged by the need to balance economic performance with environmental considerations. While prior reinforcement learning (RL) studies have incorporated carbon emissions indirectly through cost penalties or regulatory constraints, this work addresses an existing gap by treating emissions as an independent optimization objective. This study examines RL as an adaptive decision‑making approach for inventory optimization with two objectives: maximizing profit and minimizing carbon emissions. The problem is formulated as a Markov Decision Process, and four RL algorithms Proximal Policy Optimization (PPO), Phasic Policy Gradient (PPG), Advantage Actor‑Critic (A2C), and Double Deep Q‑Network (DDQN) are evaluated under identical experimental conditions. Carbon emissions are explicitly modeled in the reward function rather than embedded within operating costs. The results show that PPG achieves the highest profitability with only a modest increase in emissions, while DDQN converges faster but yields lower profit overall. Sensitivity analysis indicates that reward weighting strongly influences policy behavior, with PPO providing the most stable trade‑off between profitability and emissions.

## Introduction

Inventory management is an important part of business, as it affects many aspects like operational efficiency, cost control, and customer satisfaction. As global supply chains become more interconnected and customer expectations rise, the value of inventory models has grown significantly, enabling businesses to adapt to disruptions and optimize service levels under constraints. Effective inventory management relies on solving key trade-offs of how much and when to order, and how to allocate inventory across multiple locations or products. With the emergence of data analysis and machine learning, research has been conducted to apply these new methods to further optimize and automate inventory management. Researchers have also utilized market datasets to enhance their inventory management models, as these datasets are continuously updated to reflect the current status of global supply chains^1^.

Over the past years, supply chains have developed awareness of the adverse effects of climate change, as it creates disruptions that cause delays and raise logistic costs. As inventory management is a core part of the supply chain, this makes it a focus of the decarbonization movement. Various efforts have been undertaken to address this issue. Several policies have been developed and adopted by governments, like the limited carbon emissions policy that defines a limit to the emissions a company can produce. Another policy is carbon taxation, where companies are charged a dollar amount for every ton of emissions produced. There is also the cap-and-trade program where a set number of emissions “allowances” is issued each year and when a company exceeds its allowance, it is permitted to purchase allowances unused by other companies^2,3^.

Industries always aim to increase profitability by lowering costs, increasing efficiency, and reaching the optimum control state to meet customer demand. Focusing on sustainability has introduced another challenge for industry when optimizing their business costs. Research papers have been published trying to find the right balance between minimizing carbon emissions and reducing costs^2,4^.

According to the authors’ analysis, research gaps were identified based on the current literature. Previous reinforcement learning studies in inventory management have embedded carbon emissions indirectly within cost penalties or policy-driven constraints. Also, the few that have treated emissions as independent optimization objective didn’t use RL algorithms. Moreover, the Phasic Policy Gradient (PPG) algorithm has not been previously applied to inventory control.

This study extends the literature by (i) modeling carbon emissions as an independent optimization objective within a multi‑objective RL inventory framework, (ii) investigating the application of PPG algorithm to inventory control, and (iii) Evaluating the performance of four reinforcement learning algorithms in solving the problem.

Modelling carbon emissions as a separate reward element provides insights reveals decision-making patterns that cost-based formulations cannot capture. By isolating the environmental signal from economic pricing assumptions, the agent learns how carbon emissions influence replenishment behavior even in the absence of a carbon control policy. This modeling choice makes the profit–emissions trade‑off explicit, encourages the RL algorithms to respond to conflicting objectives, and allows better examination of policies that emerge when emissions are optimized for their own sake rather than through cost penalties.

## Literature review

This section discusses literature showing different methods of optimizing inventory systems and incorporating the reduction of carbon emissions. Optimization in inventory management involves using mathematical models, metaheuristics, and machine learning algorithms to achieve the desired goal, like minimizing costs and maximizing efficiency.

### Mathematical and heuristic models with environmental considerations

Mathematical models were first introduced by Ford W. Harris in 1913 and have since been improved to solve inventory management problems of varying levels of complexity. The emergence of non‑linear, multi‑objective, and large‑scale problems motivated the development of new methods. Heuristic and metaheuristic methods were developed to tackle these issues and resulted in satisfactory approximate solutions.

Ahmadini et al. (2021) proposed a multi-objective inventory and production model with backordering that integrated green investment decisions to mitigate environmental impact. The model aimed to maximize the profit relative to total backordered quantity, minimize holding costs, minimize total waste generated in each production cycle, and minimize the penalty costs related to green investment. They conducted a Pareto-based analysis to study the tradeoffs among the objectives and found that higher levels of green investment reduced waste and emissions while improving profits when backordering was significant. Their results showed that scenarios with greater environmental investment produced lower waste and emissions but had higher investment penalties, whereas scenarios with limited investment reduced costs but increased waste^5^.

Karampour et al. (2022) proposed a model of a bi-objective green vendor-managed inventory problem to maximize profit and minimize transportation carbon emissions using the non-dominated sorting genetic algorithm-II (NSGA-II), multi-objective of Keshtel algorithm (MOKA), and multi-objective of red deer algorithm (MORDA). Their results of their research showed that MORDA trumped both MOKA and NSGA‑II in terms of solution quality. Also, they conducted a sensitivity analysis which showed that allowing some backorders can reduce shipping frequency without eroding profitability^6^.

Pilati et al. (2024) proposed a mathematical model to solve bi-objective problems that minimize costs and reduce carbon emissions of the inventory system with perishable goods. The research paper used the Pareto front to find the best tradeoff between lowering costs and sustainability goals. The goals are anchor points representing two solutions; one is focused on reducing costs and the other reducing emissions. Their findings showed an 8% reduction in carbon emissions with an increase of 3% in costs when moving away from the anchor points^7^.

Ghosh and Mahapatra (2024) used a bi-objective optimization model that simultaneously minimizes total expected costs and emissions within an integrated supply chain with random demand, backorders, and sales losses. The authors’ model optimized variables such as order quantity, re-order level, production rate, shipment number, and vehicle velocity. The methodology employed the NSGA-II evolutionary algorithm, utilizing a Taguchi experiment for parameter tuning to identify a Pareto front of non-dominated solutions. The study concluded that while increasing vehicle velocity reduces the total expected cost, total emissions initially decreased before rising after a threshold point^8^.

### Inventory models under carbon emission policies

Zakeri et al. (2015) examined the tactical and operational planning implications of two regulatory schemes: carbon tax and cap-and-trade. The authors used a mixed-integer linear programming (MILP) model to analyze actual data from an Australian dining furniture company. Their results showed that 34% emissions reduction goal resulted in a 26.72% increase in supply chain costs. The authors concluded that while carbon trading was superior for achieving emissions reductions and maintaining service levels, carbon taxes may be preferable from an uncertainty perspective because trading costs were subject to volatile market conditions^9^.

Purohit et al. (2016) investigated the inventory lot-sizing problem within a carbon cap-and-trade system using a mixed-integer linear programming (MILP) model. The authors aimed to determine optimal replenishment timings and stock levels under non-stationary stochastic demand and cycle service level constraints. The authors found that increasing the carbon price reduced the average total cost, total inventory, and total emissions. The authors concluded that cycle service levels and demand coefficients of variation had a significant impact on performance measures regardless of demand variability^10^.

Huang et al. (2020) proposed a mathematical model using the Lagrange multiplier method to analyze how carbon emission policies affected inventory decisions, including order quantity, delivery schedules, shipment frequency, and green investment levels. The model integrated logistics decisions with policy constraints and aimed to minimize total costs subject to emission reduction constraints. They showed that policy selection depended on the efficiency of a firm’s emission reduction technology and on the rate at which the benefits from green investment diminished over time. The study found that strict emission policies worked best when companies could reduce emissions efficiently^3^.

Mishra et al. (2021) proposed a sustainable economic order quantity model (SEOQ) to exhibit a satisfactory profit for greenhouse farms under multiple cases of backorder and deterioration, using controllable carbon emissions. The authors showed that sustainable inventory management in the carbon tax and cap partial backlogging case had a better profit with the highest cycle time and the lowest value of the fraction length period, and the lowest green technology investment cost^11^.

Yadav and Khanna (2021) proposed a sustainable inventory model for perishable products that integrated expiration effects, price-sensitive demand, and a carbon-tax policy to determine the optimal selling price and replenishment cycle for maximizing total profit while minimizing carbon emissions. Their results indicated that under a carbon-tax policy, the policy achieved a better trade-off between profit and emissions than without the policy. The comparative analysis demonstrated that carbon-tax policies can reduce emissions by roughly 50% per month while decreasing profits by 7–8%^12^.

Ghosh (2022) explored the optimization of a two-echelon supply chain specifically considering the impact of random defect rates and random demand under a strict carbon cap policy. The researcher formulated a mixed-integer non-linear programming (MINLP) problem to determine the optimal production-inventory policy, where production emissions depended on the production rate and transportation emissions depended on vehicle velocity. Using Lingo optimization software, the study established a base case with an optimized production rate of 1509.12 units and a vehicle velocity of 100 km/hour, resulting in a total cost of $41,164 under a carbon limit of 600 Tons/year. The study concluded that organizations typically exhaust their allotted carbon limit to minimize costs, and that reducing lead times and defect rates were the most effective strategies for lowering total supply chain costs^13^.

### Reinforcement Learning (RL) in Inventory Management

Recent studies have applied machine learning techniques to inventory management problems, leveraging predictive models, and optimization algorithms to improve decision-making under uncertainty. However, traditional machine learning approaches often rely on static datasets and predefined rules, which limit their ability to adapt dynamically to changing environments. To overcome these limitations, researchers have turned to reinforcement learning (RL). RL has been employed to tackle different inventory management challenges, such as multi-echelon inventory optimization, multiproduct Economic Order Quantity (EOQ), multi-perishable goods, and inventory management under carbon-trading policy constraint. Reinforcement learning is a remarkable tool for decision-making because it learns from experience.

Kara and Dogan (2018) used Q-learning and Sarsa algorithms to solve an inventory management system of perishable products with random demand and deterministic lead time to minimize the total cost. The problem was solved under two replenishment policies: age-based and stock quantity. They assumed a single perishable product, while the supplier had an infinite capacity. Their results showed the effectiveness of replenishment policies that considered age information for perishable items in terms of cost optimization. Their results demonstrated that those policies, modeled using Q-learning and Sarsa algorithms, outperform traditional stock-level dependent approaches. These algorithms provided better results when demand had high variation, and products had short lifespans^14^.

Wang, Q. et al. (2022) solved a lost sales inventory system as well as a multi-echelon inventory system using double deep Q-network algorithm (DDQN). The authors compared the results in terms of demand fulfillment and cost reduction with standard algorithms like (s, S), capped base-stock policy, and constant-order policy. Their results showed that the DDQN algorithms reached better solutions than the other algorithms, with reductions in costs reaching 4%^15^.

Selukar et al. (2022) investigated the use of single-agent RL algorithms, namely Advantage Actor Critic (A2C) and Deep Deterministic Policy Gradient (DDPG), in reducing costs for the inventory management system of multiple perishable products with multiple lifetimes and inventory limitation factors. They also used models of real-world parameters such as overdue cost, shortage cost, lead time, and corruption costs. Their research showed that the single-agent RL algorithms could reduce inventory cost and spoilage rate of the goods when the products’ lead times and lifecycles were known, along with the demand distribution^16^.

Kaynov et al. (2024) used the proximal policy optimization (PPO) algorithm to solve a one-warehouse multi-retailer inventory system. The problem was solved under a lost sales case and a backordering case. Their results were compared to general-purpose rationing/allocation policies, where an improvement of 1 − 3% was made in the lost sales case, and 12 − 20% in the backordering case in terms of total expected costs^17^.

Zhou et al. (2024) proposed a multi-agent reinforcement learning model to identify cost-effective inventory policies using a modified twin delayed deep deterministic policy gradient (TD3). They introduced value decomposition and experience buffer modification to the algorithm to avoid poor performance calling it (EM-VDTD3), which resulted from random action exploration usually done in the learning process. Their results showed how the proposed method outperformed the benchmark methods regarding scalability and cost efficiency^18^.

Tian et al. (2024) proposed a deep reinforcement learning–based replenishment model called IACPPO (Integrated Advantage Actor-Critic and PPO). It integrated the Advantage Actor–Critic (A2C) and Proximal Policy Optimization (PPO) algorithms for multi-item warehouse inventory replenishment. Their approach used a continuous action space for replenishment decisions, for all items simultaneously based on historical demand, backlog and shortage costs, and inventory states. Experiments on two real-world datasets (e-commerce warehouse data and pharmaceutical sales records) showed that IACPPO achieved lower cumulative inventory costs and better average cost growth rates than forecasting models (e.g., XGBoost, GRU, LSTM, DARNN, Prophet) and several DRL baselines (DDPG, SAC, TD3, PPO, A2C, LSTM-DDPG)^19^.

### RL in inventory management with environmental considerations

Fallahi et al. (2022) integrated Q-learning with metaheuristics, particle swarm optimization, and differential evolution, to solve an EOQ problem with multiple reusable items, which reduced the environmental impact of the inventory system where the Q-learning algorithm controlled the value of the metaheuristic algorithms’ parameters. Their study demonstrated that integrating Q-learning significantly enhanced the parameter tuning of both metaheuristic algorithms, thereby contributing to improved overall optimization performance^20^.

Wang, Q. and Yang, Y. (2024) proposed a deep reinforcement learning algorithm (DRL) based on PPO Lagrange. Their algorithm controlled the ordering quantity in inventory and the carbon allowance ordering in carbon trading policy. It addressed the challenge of coordinating ordering decisions to minimize total costs while meeting emission constraints. Their results showed that their proposed method performed better than traditional inventory policies in terms of costs and resulting emissions and highlighted the criticality of carbon pricing in contracts^2^.

Wang, J. et al. (2025) proposed a proximal policy optimization (PPO) model to reduce both total costs and carbon emissions under a carbon tax policy for a multi-echelon closed-loop supply chain of waste electrical and electronic equipment. Their results showed that, compared to a traditional (s, Q) inventory policy, the PPO-based policy achieved a cost improvement of 9.1% and an emission reduction of 46.7%. The authors conducted sensitivity analysis using different carbon tax rates across scenarios with varying recycling quantities and stochastic product demand. Their results indicated that increasing the carbon tax rate generally led to lower emissions but higher operational costs, while lower tax rates reduced cost pressure but didn’t provide enough incentive for emission reduction^21^.

#### Identified research gap

Previous studies have applied machine learning methods to inventory management to address complex and multi-objective decision problems, often reporting reduced computational time compared to traditional mathematical and metaheuristic approaches. Reinforcement learning, a subset of machine learning, enables sequential decision-making under uncertainty. Despite this potential, its application to inventory management remains limited, particularly in studies that jointly consider economic and environmental objectives. Existing reinforcement learning applications have examined inventory settings such as multi-echelon systems, perishable products, and carbon trading mechanisms operating under regulatory constraints. Commonly adopted algorithms include Proximal Policy Optimization (PPO), Advantage Actor Critic (A2C), Double Deep Q Network (DDQN), and hybrid reinforcement learning models, with a primary focus on cost and service-level optimization.

The literature further indicates that studies treating carbon emissions as an independent objective in inventory management have largely relied on mathematical formulations and metaheuristic techniques. In contrast, reinforcement learning based approaches typically incorporate carbon emissions through external regulatory mechanisms, such as emission trading or taxation schemes. An analysis of reward structures in existing reinforcement learning studies shows that carbon emissions are commonly embedded within cost-related objectives, reflecting regulatory compliance. Consequently, there is limited research on reinforcement learning-based inventory optimization that models carbon emissions as a separate objective in unconstrained decision settings.

Motivated by this gap, the present study develops replenishment policies for an inventory system within an unconstrained multi-objective framework that maximizes profitability while minimizing carbon emissions. Carbon emissions are incorporated as a distinct component of the reward function, allowing direct examination of trade-offs between maximizing profits and minimizing carbon emissions without using emission policies in emissions control. The inventory problem is formulated as a Markov Decision Process, enabling reinforcement learning agents to learn adaptive ordering policies under uncertainty. Four reinforcement learning algorithms are evaluated: PPO, A2C, DDQN, and Phasic Policy Gradient (PPG). While PPO, A2C, and DDQN have been previously applied to inventory management problems, PPG is examined for the first time in this context to the authors’ knowledge. The study aims to compare the performance of these algorithms and assess their suitability for bi-objective inventory optimization.

The remainder of the paper is structured as follows. Section 3 presents the problem formulation. Section 4 describes the reinforcement learning algorithms. Section 5 reports and discusses the results. Section 6 examines the impact of reward weight selection. Section 7 highlights the managerial implications of the results. Section 8 concludes the paper.

## Formulation

This study addresses the challenge of determining replenishment policies for a single-location inventory system that maximizes profit while minimizing carbon emissions. The objective is to maximize profitability while minimizing carbon emissions generated by transportation and storage activities. To capture the nature of inventory decisions, the problem is formulated as a Markov Decision Process (MDP). The state space includes demand, inventory levels, backorders, and pending orders, while the reward function includes profit and emissions as separate elements, enabling direct trade off analysis between profit maximization and sustainability.

Demand variability is incorporated through randomization from a uniform (a, b) distribution each period. This increases the model’s robustness across multiple scenarios.

### Assumptions

Several assumptions were made to preserve the model’s practicality and maintain resemblance to real-world conditions. The assumptions are as follows:

- The problem is a single product inventory system.
- The inventory is a distribution center inventory.
- The product is sold without any modifications.
- The demand is randomly generated each time period from a uniform (a, b) distribution.
- The order quantities are batches of 100s.
- The lead time is fixed.
- The travel distance between the supplier and the warehouse is fixed.
- The same type of vehicle is used for transportation.
- The supplier has an infinite capacity.
- Unfulfilled demand is fully backordered without abandonment.

### Carbon emissions modeling

Carbon emissions are modeled directly as a separate objective of the reward function, allowing the agent to minimize emissions directly without relying on carbon emissions policy-based penalties. Emission minimization emerges from the structure of the environment: transportation emissions increase with order frequency, while holding emissions increase with average inventory levels. As a result, higher transportation or holding emissions encourage the agent to order larger quantities and maintain leaner inventory levels, whereas lower emissions allow more frequent replenishment. Through this formulation, the agent learns to balance maximizing profitability and minimizing carbon emissions by adjusting order quantity and timing in response to input parameters.

### The inventory model

The environment proceeds each time period t through the following sequence of events:

1- The orders are received from customers.

2- The demand is fulfilled entirely or partially using the current inventory.

3- The backorders are checked after the demand is fulfilled.

4- The stock is replenished according to the replenishment policy.

5- The total costs for the time period as well as the total carbon emissions are calculated.

Let the time period be $t=1,~2,~3,~...~T$. At the start of the time period, the demand (${d_t}$) and start inventory ($inv_{t}^{{start}}$) are observed, and the backordered units are tracked.

When an ordered quantity (${Q_t}$) arrives after lead time (${l_t}$) the inventory level is updated as in Eq. (1).

1 $$inv_{t}^{{start}}=inv_{{t - 1}}^{{end}}+\mathop \sum \limits_{{arrival=t}} {Q_t}$$

At the end of the time period, the inventory level ($inv_{t}^{{end}}$) and the backordered quantity (${b_t}$) are calculated as in Eq. (2) and Eq. (3).

2 $$inv_{t}^{{end}}={\mathrm{max}}\left( {inv_{t}^{{start}} - \left( {{d_t}+{b_{t - 1}}} \right),0} \right)$$

3 $${b_t}={\mathrm{max}}\left( {0,~\left( {{d_t}+{b_{t - 1}}} \right) - inv_{t}^{{start}}} \right)$$

Backorders in this model accumulate until inventory becomes available; no abandonment or lost sales effects are modeled. Any unmet demand is carried forward and fulfilled before serving new demand, and backorder cost per unit is applied each time period.

If an order is made by the algorithm, the total cost of ordering is calculated in Eq. (4).

4 $$C_{t}^{{order}}=\{ \begin{array}{*{20}{c}} {{Q_t}*{c^{unit}}+{c^{setup}},~if~{Q_t}>0} \\ {0,~otherwise} \end{array}$$

Where ($C_{t}^{{order}}$) is the total order cost for time period t, (${c^{unit}}$) is the unit purchase price, and (${c^{setup}}$) constant ordering cost of an order.

The holding cost is calculated in Eq. (5).

5 $$C_{t}^{{hold}}=\left( {\frac{{inv_{t}^{{start}}+inv_{t}^{{end}}}}{2}} \right)*{c^{hold}}$$

Where ($C_{t}^{{hold}}$) is the holding cost, and (${c^{hold}}$) is the holding cost per unit. based on the average quantity in the inventory between the start and the end of the time period.

The total cost of backorders is calculated in Eq. (6).

6 $$C_{t}^{{backorder}}={b_t}*{c^{backorder}}$$

Where ($C_{t}^{{backorder}}$) is the total backordering cost of the time period and (${c^{backorder}}$) is the backordering cost per unit.

Emissions are then calculated for order transportation and holding.

The transportation emissions are calculated in Eq. (7).

7 $$E_{t}^{{transport}}=\{ \begin{array}{*{20}{c}} {{Q_t}*{e^{transport}}*{D^{travel}}*{m^{unit}},~{Q_t}>0} \\ {0,~otherwise} \end{array}$$

Where ($E_{t}^{{transport}}$) is the total transportation emissions in kgCO_2_, (${e^{transport}}$) is constant transportation emissions rate per vehicle in kgCO_2_/T.km, (${D^{travel}}$) is the distance travelled by the transportation and it is constant in our case in km, (${m^{unit}}$) is the weight of a single unit in Tons.

The total holding emissions is calculated in Eq. (8) as the average holding emissions at the start of the time period and the end.

8 $$E_{t}^{{hold}}=\left( {\frac{{inv_{t}^{{start}}+inv_{t}^{{end}}}}{2}} \right)*{e^{hold}}+{e^{building}}$$

Where ($E_{t}^{{hold}}$) is the total holding emissions and (${e^{hold}},~{e^{building}}$) are constants for emissions of holding one unit in inventory and building emissions, respectively.

Revenue is calculated for the time period in Eq. (9).

9 $${R_t}=\hbox{min} \left( {{d_t},inv_{t}^{{start}}} \right)*\left( {1+p} \right)*{c^{unit}}$$

Where (${R_t}$) is the revenue for time period t, (p) is a constant profit margin and (${c^{unit}}$) is the unit purchase price.

The total costs ($C_{t}^{{total}}$) and total emissions ($E_{t}^{{total}}$) of each time period are calculated in Eq. (10) and Eq. (11).

10 $$C_{t}^{{total}}=~C_{t}^{{order}}+C_{t}^{{hold}}+C_{t}^{{backorder}}$$

11 $$E_{t}^{{total}}=E_{t}^{{transport}}+E_{t}^{{hold}}$$

Then the profit for the time period is calculated in Eq. (12).

12 $${P_t}={R_t} - C_{t}^{{total}}$$

The state space (${s_t}$) includes the necessary information for the agent to make a well-informed decision. The state space contains the current demand (${d_t}$), the current inventory level ($inv_{t}^{{start}}$), the backordered quantity (${b_t}$), the total amount ordered pending ($Q_{t}^{{pend}}$), and the lead time (${l_t}$). So, the state space is set to be.

13 $${s_t}=\left[ {{d_t},inv_{t}^{{start}},{b_t},Q_{t}^{{pend}},{l_t}} \right]$$

The agent is set to make order quantities (${Q_t}$) in batches with size 100 units, reflecting realistic batch ordering in supply chains. The action space is a set of discrete actions that range from zero to the maximum capacity of the inventory in increments of 100. To make the number of discrete order actions (${n^{action}}$) dependent on the maximum capacity in Eq. (14).

14 $${n^{action}}=\left( {\frac{{in{v^{max}}}}{{100}}} \right)+1$$

Where $in{v^{max}}$ is the maximum capacity of the inventory. So, the action space (a) in Eq. (15) is set to be.

15 $${a}=\left[ {{Q_t}} \right]$$

During model development, the agent occasionally generated order quantities exceeding the inventory capacity, leading to overflow. To address this, we implemented an action-masking mechanism that restricts the agent’s available actions. The possible actions are limited to the available space ($in{v^{available}}$) in inventory. Equation (16) is the equation for action masking.

16 $${n^{max~action}}=\left( {\frac{{in{v^{available}}}}{{100}}} \right)$$

Where only the first (${n^{max~action}}$) actions are permitted at time t.

The reward function aims to balance costs and emissions reduction. To prevent the agent from going towards trivial solutions, an incentive in the form of revenue has been added to encourage the agent to try to find out realistic ordering policies. The reward function is composed of a Profit term (${P_t}$), and Emissions term ($E_{t}^{{total}}$). The reward function is shown in Eq. (17).

17 $$r=\mathop \sum \nolimits_{{t=1}}^{T} \left( {{w^{profit}} \cdot {P_t} - {w^{emission}} \cdot E_{t}^{{total}}} \right)$$

Where (${w^{profit}}$, ${w^{emission}}$) are weights to balance each part of the reward function. This function allows adjusting the tradeoff between economic and sustainable performance through weights.

While the problem involves multiple performance criteria, we adopt a weighted sum scalarization of profit and emissions to construct a single scalar reward at each decision step. This approach combines the criteria into a unified objective, enabling the application of standard single-objective reinforcement learning algorithms while still capturing the trade-off between economic performance and environmental impact.

## Benchmark RL algorithms

**Fig. 1:**
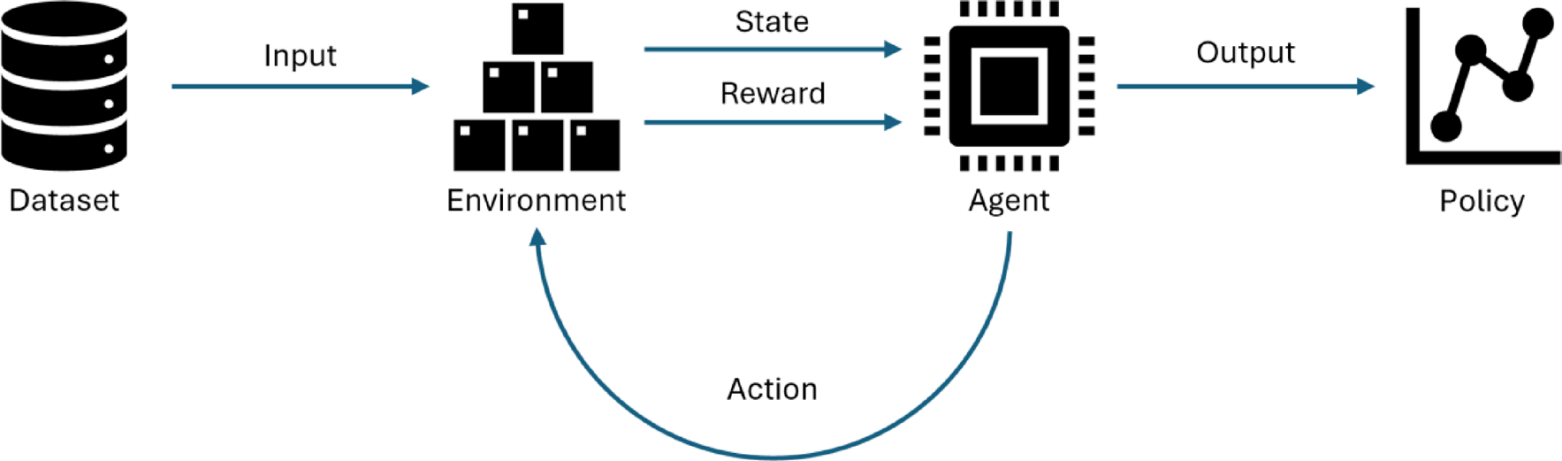
Illustration of how the RL algorithm interacts with the environment and converges into an effective ordering policy.

This section discusses the RL algorithms used in this study. Figure 1 illustrates how the RL agent interacts with the environment and converges into a suitable policy to solve the problem. The agent receives the system state, selects replenishment actions based on the available actions, and then receives feedback in the form of a reward. Through repeated interaction, the agent iteratively improves its decision-making policy and converges toward a policy that balances the two objectives.

The following subsections will discuss the 4 RL algorithms in this study: Proximal Policy Optimization (PPO), Phasic Policy Gradient (PPG), Advantage Actor-Critic (A2C), and Double Deep Q-Network (DDQN).

### PPO

PPO is a policy-gradient algorithm introduced in 2017 by Schulman et al.^22^. It is widely adopted and reliable policy-gradient algorithms in reinforcement learning. It improves training stability by constraining policy updates, instead of allowing large and potentially destructive gradient steps. PPO achieves this using a clipping mechanism to keep the new policy close to the old one. The clipping equation:

18 $${L^{CLIP}}\left( \theta \right)={E_t}\left[ {min\left( {{r_t}\left( \theta \right){A_t},clip\left( {{r_t}\left( \theta \right),1 - \epsilon ,1+\epsilon } \right){A_t}} \right)} \right]$$

Where ${r_t}\left( \theta \right)$ is the ratio between the probabilities of the old and the new policies, and $\varepsilon$ is a small number used for clipping.

PPO is chosen because it is commonly used for both discrete and continuous tasks, offering a good balance of stability and performance.

### PPG

PPG was introduced in 2020 by Cobbe et al.^23^ separates policy and value training into distinct phases, improving sample efficiency and reducing interference. Its joint loss function balances policy and auxiliary value training:

19 $$L\left( \theta \right)={L_\pi }\left( \theta \right)+\beta {L^{aux}}\left( \theta \right)$$

where ${L_\pi }$ is the policy loss, ${L^{aux}}$ is the auxiliary phase loss for value estimation, and $\beta$ controls their trade-off.

PPG was chosen as a recent advancement in policy-gradient methods. It is relevant because its two‑phase structure separates policy optimization from value function refinement reducing interference between learning signals. This is useful in the scalarized multi‑objective reward setting, where profit and emissions can exert conflicting gradients. PPG’s auxiliary phase helps stabilize learning when such heterogeneous reward components are present.

### A2C

A2C is a synchronous version of asynchronous advantage actor-critic (A3C) using multiple learners to stabilize gradient estimates without asynchronous execution^24^. A2C combines policy optimization with a baseline to reduce variance. The actor update is based on the advantage function:

20 $$A\left( {{s_t},{a_t}} \right)=Q\left( {{s_t},{a_t}} \right) - V\left( {{s_t}} \right)$$

The policy loss is defined as:

21 $${L_\pi }\left( \theta \right)={E_t}\left[ {log{\pi _\theta }\left( {{a_t}\mid {s_t}} \right)A\left( {{s_t},{a_t}} \right)} \right]$$

A2C provides a simpler and more computationally efficient actor-critic baseline, serving as a comparison point for advanced policy-gradient methods.

### DDQN

DDQN extends the Deep Q-Network (DQN) by introducing a second network to decouple action selection from evaluation, thereby reducing the overestimation bias observed in standard Q-learning^25^. DDQN addresses the overestimation bias in Q-learning by decoupling action selection from evaluation. Its update rule is:

22 $${y_t}={r_{t+1}}+\gamma Q\left( {{s_{t+1}},argmaxQ\left( {{s_{t+1}},a;{\theta _t}} \right),\theta _{t}^{\prime }} \right)$$

where ${\theta _t}$ are online network parameters and $\theta _{t}^{\prime }$ are target network parameters.

## Results and discussion

In this research, we simulated an inventory management system designed to increase profitability while minimizing carbon emissions. Backorders are used to account for unfulfilled demand for a penalty, and the wait time is indefinite. We compared four different RL algorithms: PPO, PPG, A2C and DDQN. The algorithms were trained and validated using the same training parameters to ensure a fair comparison.

Demand in the simulation was randomly generated each time period from a Uniform (a, b) distribution. The rest of the environment parameters were: the holding cost per unit, the unit purchase price, the unit weight, the order constant cost, the warehouse building emissions, the holding emission rate, the transport vehicle emissions rate, the travel distance, the lead time, which in this case was a constant across the time frame of training, and the backorder cost. The numerical values of environment parameters are shown in Table 1.

**Table 1:** Environment parameters.

Parameter	Symbol	Value	Unit
Max inventory capacity	$in{v^{max}}$	3000	Units
Demand data lower bound	${d^{min}}$	0	Units
Demand data upper bound	${d^{max}}$	1500	Units
Profit parameter weight	${w^{profit}}$	0.5	
Emissions parameter weight	${w^{emission}}$	0.5	
Holding cost per unit	${c^{hold}}$	0.1	Cost Unit
Unit purchase price	${c^{unit}}$	100	Cost Unit
Unit weight	${m^{unit}}$	0.01	Tons
Order constant Cost	${c^{setup}}$	1000	Cost Unit
Backorder cost per unit	${c^{backorder}}$	10	Cost Unit
Warehouse building emissions	${e^{building}}$	9.17	kgCO_2_
Holding emission rate	${e^{hold}}$	0.00174	kgCO_2_/unit
Transport vehicle emissions rate	${e^{transport}}$	0.336	kgCO_2_/T.km
Travel distance	${D^{travel}}$	50	Km
Lead time	${l_t}$	3	Time unit

**Fig. 2:**
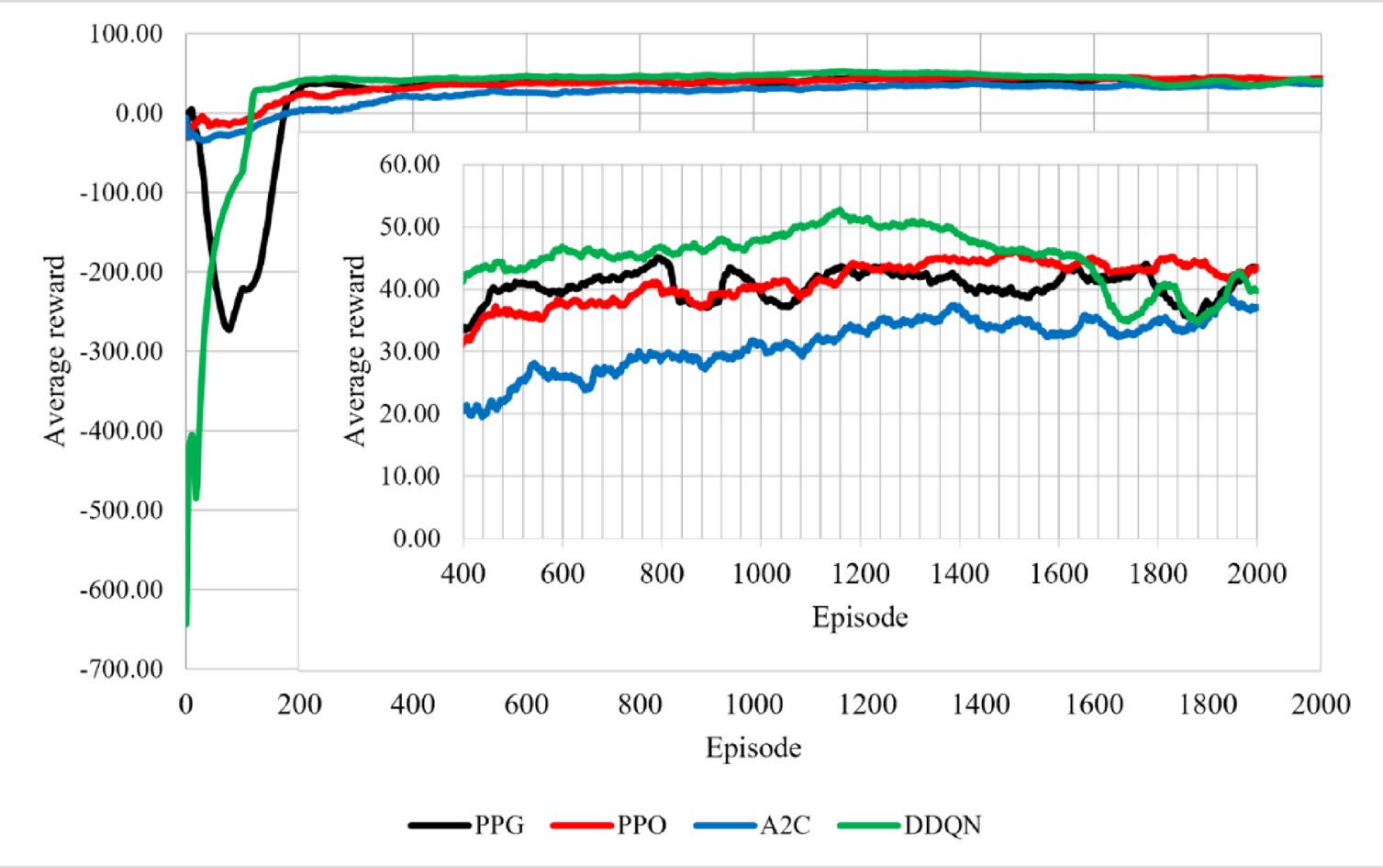
Learning curves (reward vs. episode) for all agents. Curves show a 100-episode running average rewards.

**Fig. 3:**
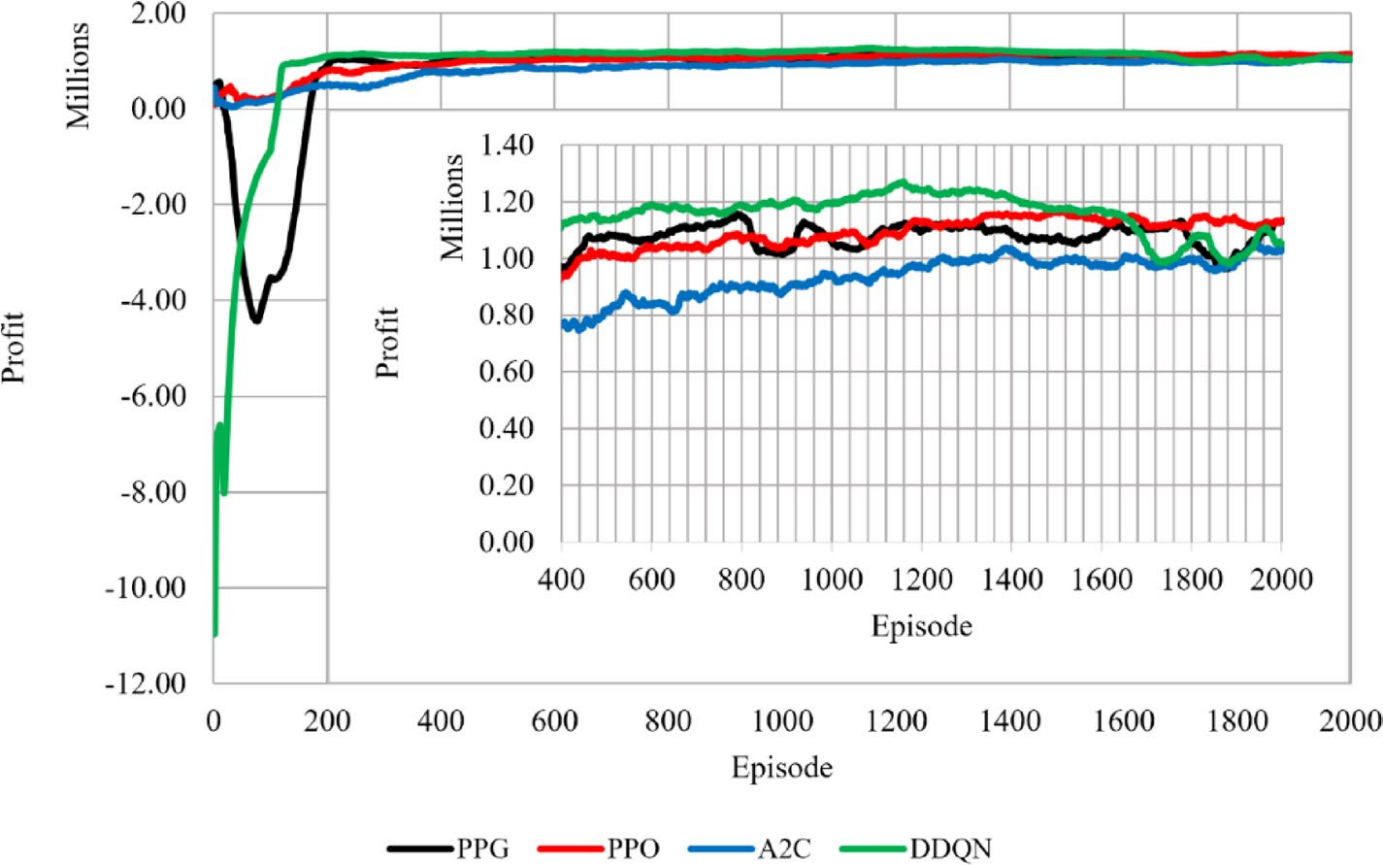
The change in total profit during training.

**Fig. 4:**
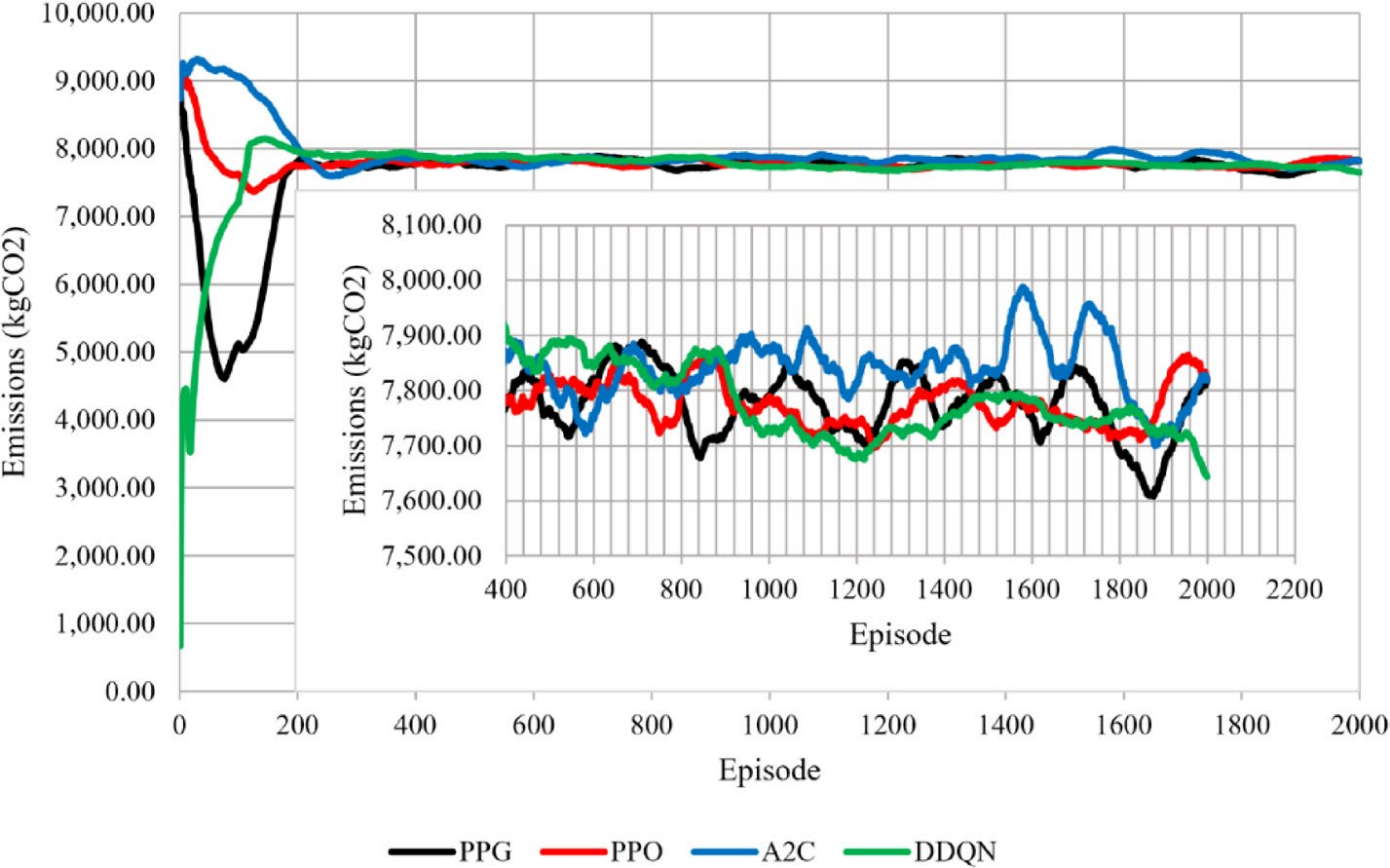
The change in total emissions (kgCO_2_) during training.

During experimentation we set the learning rate that is most suitable for each RL algorithm, based on settling speed as well as highest validation score over three training runs. On the training data, we trained each RL algorithm and adjusted the key parameters, which are shown in Table 2. On the test data, we evaluated each RL algorithm by calculating the cumulative profit and the total carbon emissions. The experimental environment in this research included a 64-bit Windows 11 operating system, (AMD) Ryzen 5 3600, CPU @4.00 GHz, (Nvidia) GTX 1660 Super, GPU @6 GB GDDR6 memory, 16 GB of RAM, and PyTorch 2.4.0., the profit and emissions dynamics each episode were compared in a line graph, as shown in Figs. 3 and 4. As for the learning curve highlighting convergence patterns. We generated a scatterplot graph for each agent and used a 100-episode running average to trends as shown in Fig. 2. For visual clarity, Figs. 2, 3 and 4 display a representative single‑seed trajectory; the variability across independent runs is quantified in Tables 3 and 4.

**Table 2:** Hyperparameters used during training and validation.

Parameter	Symbol	PPO	PPG	A2C	DDQN
Learning rate	$lr$	1e-4	3e-4	1e-4	3e-4
Discount factor	$\gamma$	0.99	0.99	0.99	0.99
GAE lambda	${\lambda ^{GAE}}~$	0.9	0.9	0.9	NA
Entropy coefficient	$\alpha$	0.005	0.01	0.02	NA
Clipping factor	$\epsilon$	0.2	0.2	NA	NA
No. of epochs	${n^{epochs}}$	10	Policy = 10 Auxiliary = 6	10	NA
Neural network width		256	256	64	128
Network depth		Actor: 3 Critic: 3	Actor: 3 Critic: 3	Actor: 3 Critic: 4	3
Activation function		ReLU	ReLU and Softmax	ReLU and Softmax	ReLU and Linear output
Optimizer		Adam	Adam	Adam	Adam
Actor critic structure		Shared	Separate	Shared	NA
Batch size		256	256	NA	64
Number of training episodes	${n^{episodes}}$	5000	5000	5000	5000

Table 3 provides the per seed results for each algorithm, including settling episodes, average profit, and average emissions, allowing comparison of variability across independent runs. Note that an agent ‘settled’ when the absolute change in the average reward was < 5% for 100 consecutive episodes. The average profit and emissions in the table were taken after episode 2000 to ensure that the numbers reflect the average profit and emissions after settling.

**Table 3:** Summary statistics of training seeds for the agents.

Seed	Particular	PPO	PPG	A2C	DDQN
Run 1					
	Episodes till settling	1700	1400	1000	500
	Average profit	$$\begin{gathered} 1,072,370~ \hfill \\ \pm 196,277 \hfill \\ \end{gathered}$$	$$\begin{gathered} 1,145,323 \hfill \\ \pm 146,775 \hfill \\ \end{gathered}$$	$$\begin{gathered} 1,067,392~~ \hfill \\ \pm 166,003 \hfill \\ \end{gathered}$$	$$\begin{gathered} 962,277 \hfill \\ \pm ~197,053 \hfill \\ \end{gathered}$$
	Average emissions	$7,725 \pm 202$	$7,741 \pm 169$	$7,813 \pm 212$	$7,536 \pm 201$
Run 2					
	Episodes till settling	800	1100	1800	700
	Average profit	$$\begin{gathered} 995,031 \hfill \\ \pm 350,348 \hfill \\ \end{gathered}$$	$$\begin{gathered} 1,147,346 \hfill \\ \pm 141,841 \hfill \\ \end{gathered}$$	$$\begin{gathered} 1,051,502 \hfill \\ \pm 175,171 \hfill \\ \end{gathered}$$	$$\begin{gathered} 1,025,449 \hfill \\ \pm 213,419 \hfill \\ \end{gathered}$$
	Average emissions	$7,663 \pm 237$	$7,748 \pm 178$	$7,793 \pm 198$	$7,629 \pm 236$
Run 3					
	Episodes till settling	600	1400	1300	700
	Average profit	$$\begin{gathered} 997,677 \hfill \\ \pm 271,409 \hfill \\ \end{gathered}$$	$$\begin{gathered} 1,133,753 \hfill \\ \pm 139,327 \hfill \\ \end{gathered}$$	$$\begin{gathered} 880,101 \hfill \\ \pm 572,159 \hfill \\ \end{gathered}$$	$$\begin{gathered} 987,683 \hfill \\ \pm 177,684 \hfill \\ \end{gathered}$$
	Average emissions	$7,662 \pm 203$	$7,761 \pm 176$	$7,808 \pm 386$	$7,670 \pm 182$

Figures 2, 3 and 4 show training till episode 2000 only since it was noticed that beyond that the change in the running average reward is less than 1%. Figure 2 shows the learning behavior of the learning (RL) agents during training. The PPO and A2C algorithms exhibited relatively similar convergence patterns. PPG showed weaker exploration performance compared to the other agents characterized by initial fluctuations during the exploration phase before stabilizing. DDQN had the best training performance as it settled at a faster rate than the other agents. DDQN begins with lower initial reward values, likely due to epsilon‑greedy exploration, but converges faster than the other agents. Figures 3 and 4 provide additional insight into the agents’ performance in terms of profit and emissions.

Below in Table 4 is a summary of the settling episodes, profit and emissions of the four agents. The results show that PPG profit confidence interval (CI) interval lies above DDQN, indicating a clear profitability advantage for PPG over DDQN from the training results. In contrast, PPG and PPO profit CI intervals slightly overlap, however PPG can achieve higher profits. For carbon emissions, A2C CI interval is higher than PPG, indicating higher emissions for A2C relative to PPG, while PPO and DDQN show lower emissions ranges with overlapping intervals. Overall, these intervals confirm the previously mentioned results: PPG delivers the highest profitability with a slight emissions increase, PPO offers a balanced trade‑off, DDQN converges fastest but to lower profits, and A2C exhibits higher emissions on average.

**Table 4:** Summary statistics for the agents across the training seeds.

Particular	PPO	PPG	A2C	DDQN
Average settling episode	$1,033 \pm 586$	$1,300 \pm 173$	$1,367 \pm 404$	$633 \pm 115$
Average profit	$$\begin{gathered} 1,021,693 \hfill \\ \pm 43,908 \hfill \\ \end{gathered}$$	$$\begin{gathered} 1,142,141 \hfill \\ \pm 7,334 \hfill \\ \end{gathered}$$	$$\begin{gathered} 999,665 \hfill \\ \pm 103,850 \hfill \\ \end{gathered}$$	$$\begin{gathered} 991,803 \hfill \\ \pm 31,787 \hfill \\ \end{gathered}$$
95% CI	[912,619, 1,130,765]	[1,123,921, 1,160,359]	[741,687, 1,257,642]	[912,839, 1,070,766]
Average emissions	$7,683 \pm 36$	$7,750 \pm 10$	$7,805 \pm 10$	$7,612 \pm 69$
95% CI	[7,593, 7,772]	[7,724, 7,775]	[7,778, 7,830]	[7,441, 7,782]

To examine environmental robustness, we repeat the training with a randomized demand from Poisson distribution (Lambda = 750) that preserves the baseline mean while reducing variance. As shown in Table 5 below, DDQN settles fastest on average, followed by PPO, A2C, and PPG reflecting the advantage of value-based learning when dynamics are stable. In terms of profitability, PPG remains the leading algorithm with an average profit of 1,349,933 and a tight 95% CI, whereas DDQN surpasses PPO in this low variance environment (1,233,031 vs. 1,185,941), reflecting DDQN’s advantage when demand becomes more predictable. Emissions remain tightly grouped, with PPG showing the lowest average (8,141 kgCO_2_), followed by A2C (8,159), PPO (8,258), and DDQN (8,301). The consistency of algorithm differences within the Poisson confidence intervals confirms that the comparative conclusions identified under uniform demand: PPG as the profit‑efficient leader, and DDQN as the fastest to converge.

**Table 5:** Summary statistics across training seeds with Poisson distribution.

Particular	PPO	PPG	A2C	DDQN
Average settling episode	$767 \pm 115$	$1,267 \pm 208$	$1,000 \pm 200$	$700 \pm 520$
Average profit	$$\begin{gathered} 1,185,941 \hfill \\ \pm 17,767 \hfill \\ \end{gathered}$$	$$\begin{gathered} 1,349,933 \hfill \\ \pm 11,730 \hfill \\ \end{gathered}$$	$$\begin{gathered} 1,274,456 \hfill \\ \pm 4,626 \hfill \\ \end{gathered}$$	$$\begin{gathered} 1,233,031 \hfill \\ \pm 126,984 \hfill \\ \end{gathered}$$
95% CI	[1,141,804, 1,230,077]	[1,320,794, 1,379,072]	[1,262,963, 1,285,947]	[917,585, 1,548,475]
Average emissions	$8,258 \pm 70$	$8,141 \pm 11$	$8,159 \pm 2$	$8,301 \pm 239$
95% CI	[8,084, 8430]	[8,113, 8168]	[8,153, 8164]	[7,706, 8895]

## Sensitivity analysis

In this section, we experimented with different weights on the profit and emissions elements of the reward function to examine how the agent’s ordering policy changes. We constrained the weights so that their sum equal to 1, as in Eq. (24).

24 $${w^{profit}}+~{w^{emission}}=1$$

We trained the agents on 5 cases, which are presented in Table 6. One case is balanced, while two favor increasing profit, and two favor minimizing emissions. Each case was trained for an equal number of times and evaluated using the same validation data after each individual training. The purpose was to capture the difference in behavior and help interpret the results.

**Table 6:** Weights of the profit and emissions components of the reward function.

No.	Case	${w^{profit}}$	${w^{emission}}$
1.	Profit extreme bias	0.9	0.1
2.	Profit bias	0.7	0.3
3.	Balance	0.5	0.5
4.	Emissions bias	0.3	0.7
5.	Emissions extreme bias	0.1	0.9

**Fig. 5:**
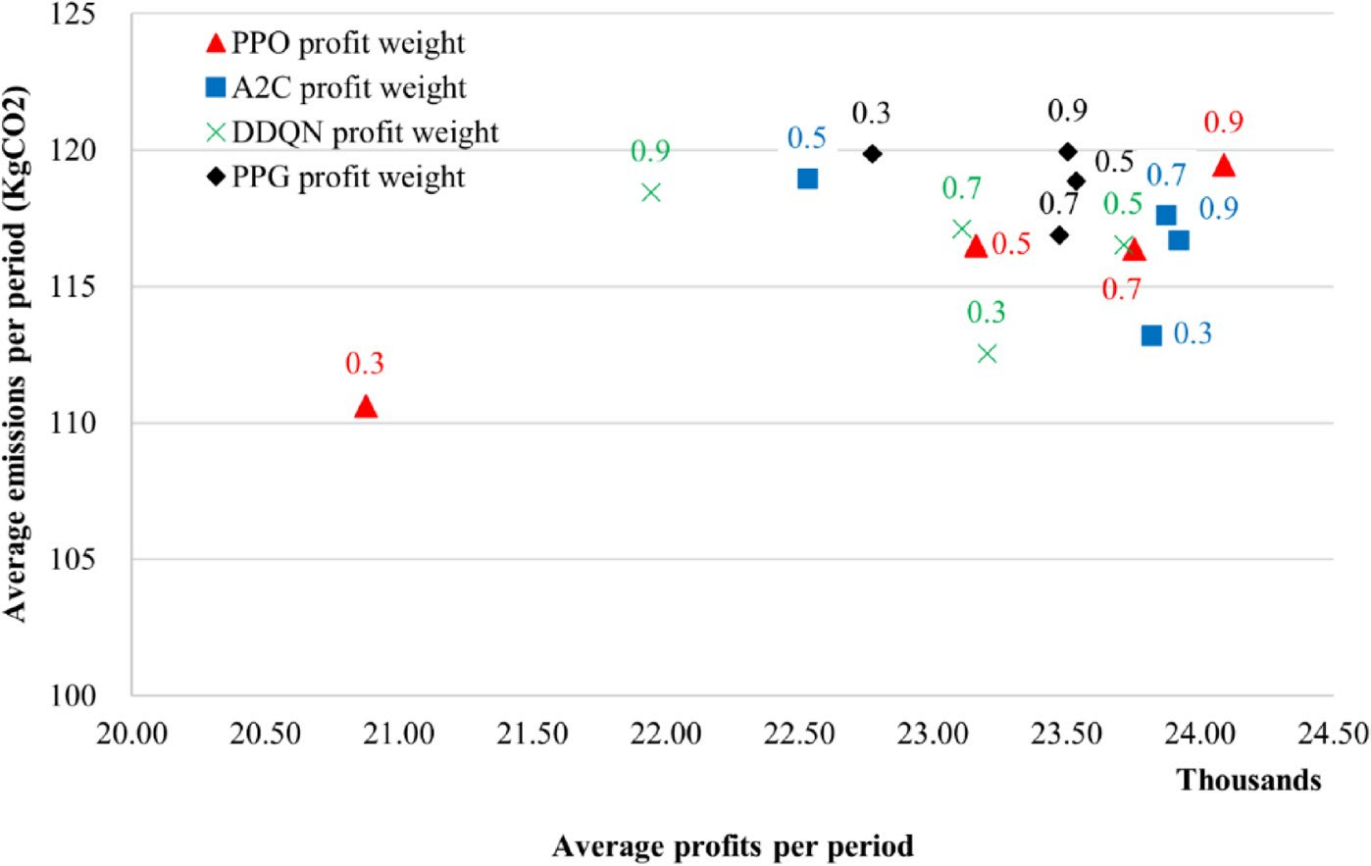
Validation profit and emissions for all algorithms under different profit/emissions weight configurations.

Figure 5, shows that each algorithm responds differently to changes in the profit and emissions weights. For the extreme emissions bias case, all agents learned near-zero ordering policies resulting in losses. This reflects a structural limitation of the reward design, in which extreme emissions dominant weights suppress economic incentives and drive the agent toward trivial solutions. Because the curves were visually flat, they are omitted from Fig. 5. When studying the agents’ behavior, we found that PPO was the most stable out of the four algorithms. As expected, increasing the profit weight increased average profit, whereas increasing the emissions weight reduced profit. When studying each weights case individually, we found that DDQN had better results in terms of emissions, PPG was clustered in one place and didn’t show considerable sensitivity when changing weights. A2C similar to PPG, however it managed to achieve better results especially in case 2. PPO was moderate but had the most uniform and consistent performance and achieved the highest average profit in case 5. The performance of PPO relative to A2C matches the comparison result of Tian, R et al. (2024)^19^ in terms of difference in performance even though the reward function is different.

**Fig. 6:**
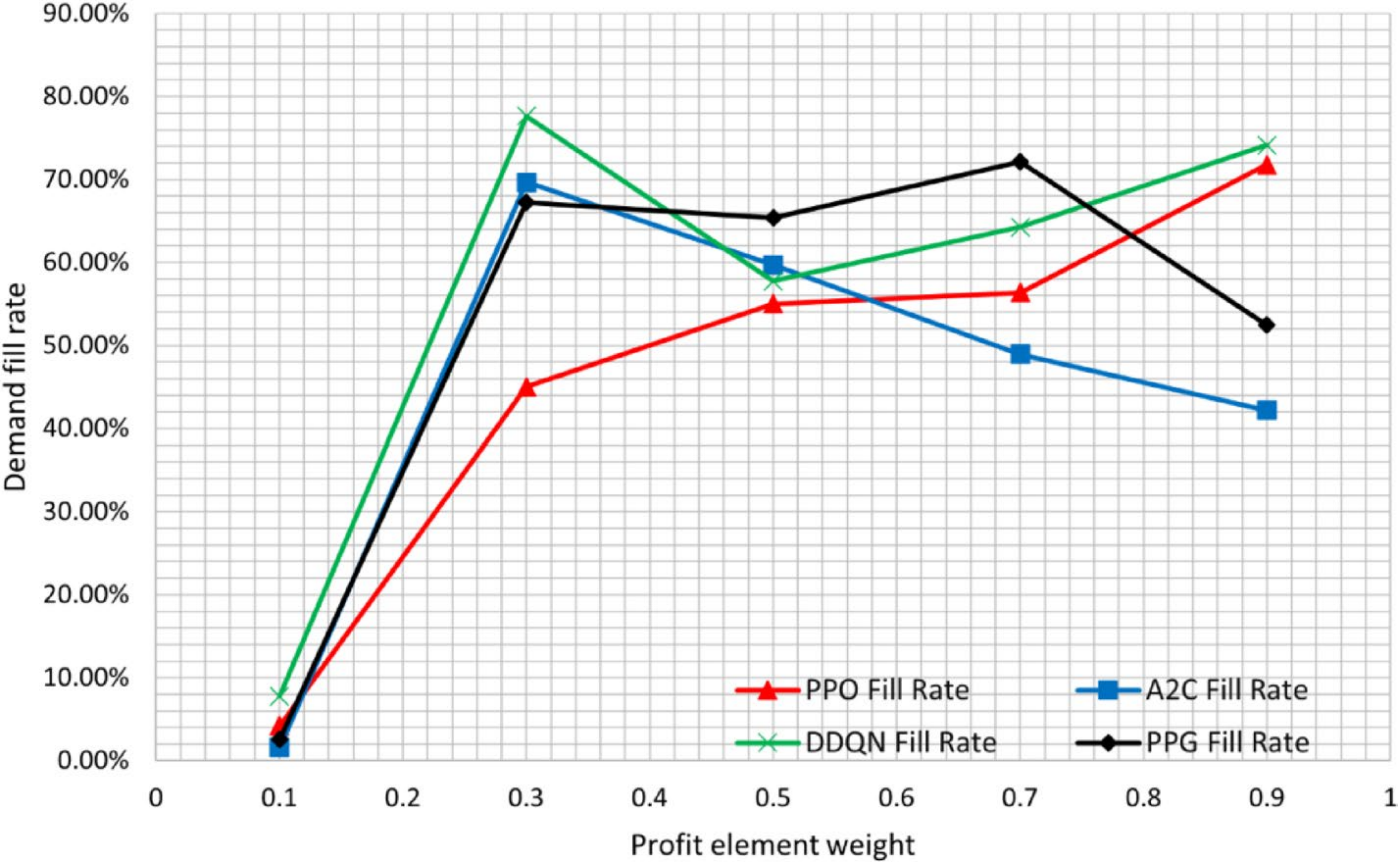
Demand fill percentage for each case. This shows the average demand fulfillment for each algorithm each time period at different biases.

Figure 6 shows more insight using the average order fulfillment percentage as a metric across time periods under varying configurations of the reward function weights. The PPO algorithm showed the most consistent performance, with a uniform fill rate curve that reflected stable sensitivity to changes in the profit-emissions tradeoff. DDQN showed less stability, with a higher fill rate in case 2. In contrast, both PPG and A2C showed irregular patterns, with fill rates fluctuating across scenarios, indicating limited responsiveness to changing weights.

## Managerial implications

The findings of this study offer actionable insights for managers seeking to balance profitability and sustainability in inventory systems. RL provides a dynamic alternative to traditional inventory models by enabling adaptive decision-making under uncertainty. However, selecting the appropriate RL algorithm and configuring reward weights are critical to achieving desired outcomes.

To translate the learned policies into practical inventory behavior, Table 7 shows operational metrics for the balanced case. The results show distinct behavioral profiles for each algorithm. PPG achieves the highest profitability with a relatively moderate average on hand inventory and backlog, reflecting efficient stock utilization. PPO provides the most balanced and consistent operational performance, with a stable fill rate and controlled ordering quantity. A2C shows higher backlog and emissions, consistent with its tendency towards less optimal replenishment timing. DDQN places smaller orders, which is reflected in its ordering quantity and fill‑rate pattern.

**Table 7:** Validation operation metrics for balanced case (${w^{profit}}$= 0.5 | ${w^{emission}}$*=*0.5).

Particular	PPO	PPG	A2C	DDQN
Average backordered Quantity	608 ± 300	385 ± 61	483 ± 217	467 ± 86
Average ordered quantity per period	631 ± 23	644 ± 18	645 ± 15	630 ± 26
Average on hand inventory	1,096 ± 83	1,162 ± 145	1,106 ± 145	1,224 ± 82
Average fill rate %	55.02 ± 11.59	64.46 ± 3.83	60.17 ± 10.09	59.99 ± 4.26

### Algorithm selection guidance

- **PPG** is recommended for organizations prioritizing profitability while maintaining sustainability, as it achieved the highest profit with only a limited increase in emissions. Managers may prefer to keep the reward setup in the balance case (“Case 3”).
- **PPO** is suitable for managers seeking a stable and balanced trade-off between economic and environmental objectives, particularly under varying reward configurations.
- **DDQN** is ideal for scenarios requiring rapid deployment and fast convergence, though managers should be cautious about its less reliable profitability performance.
- **A2C** serves as a computationally efficient baseline for simpler systems or resource-constrained environments. Managers may prefer to make the algorithm biased towards profitability objective for better results.

### Reward weight configuration

Sensitivity analysis shows that adjusting profit and emissions weights significantly influences policy behavior. Managers can use this flexibility to align RL policies with organizational priorities without relying on external regulatory constraints.

## Conclusion

This research investigates reinforcement learning (RL) for multi-objective inventory optimization, explicitly modeling profitability and carbon emissions as separate objectives. The problem was formulated as a Markov Decision Process (MDP), and four RL algorithms Proximal Policy Optimization (PPO), Phasic Policy Gradient (PPG), Advantage Actor-Critic (A2C), and Double Deep Q-Network (DDQN) were evaluated under identical conditions.

PPG achieves the highest profitability with only a limited increase in carbon emissions. PPO came second in terms of profits with a slight decrease in emissions. DDQN exhibited the fastest convergence but made the least profits. A2C provided moderate profit but had the highest carbon footprint. These findings are supported by the multi‑seed results in Table 3 and the 95% confidence intervals in Table 4. Testing under Poisson demand further supports the consistency of the comparative findings with minor differences such as PPO performing worse than DDQN; however PPO is more stable as shown in the 95% CI in Table 5.

Sensitivity analysis revealed that reward weight adjustments significantly influence policy behavior, providing actionable insights for managers seeking to align operational efficiency with sustainability priorities. PPO showed more stability than the other algorithms with different weight configurations.

Limitations to the developed model relies on simplifying assumptions that facilitate controlled experimentation. These include the use of uniform demand, fixed lead time, constant transportation distance and emission factors, batch ordering in fixed increments, infinite supplier capacity, and full backordering without abandonment. Such assumptions don’t reflect real world variability such as stochastic lead times, fluctuating transportation emissions, finite supplier constraints, and partial backordering. In addition, the scalarized reward formulation makes the learned policies dependent on the selected weight vector and may not explore non‑convex regions of the profit–emissions trade‑off, meaning the solutions do not represent the full Pareto set. Finally, extreme reward weight settings induces trivial policies such as the case of extreme emissions bias due to the profit incentive signal being weak to influence behavior.

Future research can incorporate empirical demand distributions, variable lead times, dynamic emission factors, alternative backordering mechanisms, finite‑capacity supply, and explore Pareto frontier. Future research can also extend this framework to multi-echelon and multi-product systems. These directions will enhance both theoretical understanding and practical applicability, supporting the development of intelligent inventory policies that advance profitability and sustainability in modern supply chains.

## Data Availability

The data used for this study can be provided upon request.The emissions data for the holding (operations) and the fixed building emissions, as well as transportation emissions, were obtained from the Ecoinvent database^1^. The database can be accessed through the following link: https://ecoinvent.org/.

## Abbreviations

A2C: Advantage Actor Critic
CI: Confidence Interval
DDQN: Double Deep Q Network
DRL: Deep Reinforcement Learning
EOQ: Economic Order Quantity
MDP: Markov Decision Process
PPG: Phasic Policy Gradient
PPO: Proximal Policy Optimization
RL: Reinforcement Learning
TD3: Twin Delayed Deep Deterministic Policy Gradient

## List of symbols

a: Action space
T: Max time period (Time unit
t: Time period (Time unit
${b_t}$: Backordered quantity at time period *t* (Units)
${c^{backorder}}$: Backordering cost per unit (Cost unit/unit)
${c^{hold}}$: holding cost per unit (Cost unit/unit)
${c^{setup}}$: Constant order cost (Cost unit)
${c^{unit}}$: Purchase price per unit (Cost unit/unit)
$C_{t}^{{hold}}$: Total holding cost at time period *t* (Cost unit)
$C_{t}^{{backorder}}$: Total backordering cost at time period *t* (Cost unit)
$C_{t}^{{order}}$: Total ordering cost at time period *t* (Cost unit)
$C_{t}^{{total}}$: Total costs at time period *t* (Cost unit)
${d_t}$: Demand at time period *t* (Units/Time)
${D^{travel}}$: Distance travelled by transportation in km (km)
${e^{hold}}$: Constant holding emissions rate per unit (kgCO_2_)
${e^{building}}$: Constant building emissions rate per unit (kgCO_2_)
${e^{transport}}$: Constant transportation emissions rate per vehicle (kgCO_2_/T.km)
$E_{t}^{{hold}}$: Total holding carbon emissions at time period *t* (kgCO_2_/Time unit)
$E_{t}^{{total}}$: Total carbon emissions at time period *t* (kgCO_2_/Time unit)
$E_{t}^{{transport}}$: Total transportation carbon emissions at time period *t* (kgCO_2_/Time unit)
$in{v^{available}}$: Available inventory space (Units)
$in{v^{max}}$: Maximum inventory capacity (Units)
$inv_{t}^{{end}}$: Inventory level at the end of the time period *t* (Units)
$inv_{t}^{{start}}$: Inventory level at the start of the time period *t* (Units)
${l_t}$: Lead time at time period *t * (Time unit)
${m^{unit}}$: Mass of each unit (Tons)
${n^{action}}$: Number of actions
${n^{max~action}}$: Maximum number of actions allowed
p: Profit margin (Percentage)
${P_t}$: Total profit at time period *t* (Cost unit)
${Q_t}$: Order quantity at time period *t* (Unit)
$Q_{t}^{{pend}}$: Total amount ordered pending at time period t (Units)
${R_t}$: Total revenue at time period *t* (Cost unit)
${s_t}$: State space at time period *t*
${w^{emission}}$: Weight of the emissions reward element
${w^{profit}}$: Weight of the profit reward element

## Code availability

The code used to implement the environment, training, and evaluation in this study is available from the authors upon request. All parameters used to generate Tables 3, 4, 5, 6 and 7 are available in Tables 1 and 2.
